# Society Meetings

**Published:** 1898-10

**Authors:** 


### SOCIETY MEETINGS.

---

The American Association of Obstetricians and Gynecologists held its eleventh annual meeting at Pittsburg, Pa., September 20-22, 1898. The attendance was large and the discussions spirited and instructive.

The next meeting was appointed to be held at Indianapolis, Ind., September, 1898.

The local committee of arrangements, headed by Dr. John Milton Duff, as chairman and Dr. William J. Asdale, as secretary, through their energy and good judgment contributed very largely to the success of the meeting. The complimentary banquet tendered by the trustees and faculty of the Western Pennsylvania Medical College, on the evening of the second day, was a feast worthy of the host and appreciated by the guests. The menus, gastronomic and psychic, were most elaborate in design as well as artistic in service—the one most satisfying physically and the other a charm mentally.

The following-named officers were elected: President, Edward J. Ill, M. D., Newark, N. J.; vice-presidents, Edwin Ricketts, M. D., Cincinnati, O., and A. B. Miller, M. D., Syracuse, N. Y.; secretary, William Warren Potter, M. D., Buffalo, N. Y.; treasurer, X. O. Werder, M. D., Pittsburg, Pa.; executive council, A. Vander Veer, M.D., Albany, N. Y.; L. S. McMurtry, M. D., Louisville, Ky.; W. E. B. Davis, M. D., Birmingham, Ala.; John Milton Duff, M. D., Pittsburg, Pa.; L. H. Dunning, M. D., Indianapolis, Ind., and Walter B. Chase, M. D., New York.

---

The Buffalo Academy of Medicine held meetings during the month of September, 1898, as follows:

*Section on Surgery.*—Tuesday evening, September 6th, program: Duties of medical officers during active campaigning, Dr. Wm. Warren Potter, Brevet Lieut.-Col., U. S. V.; Coöperation of physician and surgeon in abdominal cases, Dr. A. L. Benedict. Discussion by Drs. Frederick, Park, Mann and Congdon.

*Section on Medicine.*—Tuesday evening, September 13th, program: Chronic joint affections, commonly called rheumatism, Dr. Charles G. Stockton; Rheumatism of childhood, Dr. Benjamin G. Long.

*Section on Pathology.*—Tuesday evening, September 20th, program: Convicts' skulls and brains, Dr. M. V. Ball, of Warren, Pa. Exhibition of specimens.

*Section on Obstetrics.*—Tuesday evening, September 27th, program: Some common mistakes in gynecology, by Dr. M. D. Mann; Discussion led by Dr. Frederick.

The Tri-state Medical Society, of Alabama, Georgia and Tennessee, will hold its tenth annual meeting at Birmingham, Ala., Tuesday, Wednesday and Thursday, October 25, 26, 27, 1898, under the presidency of James A. Goggans, M. D., of Alexandra City, Ala. The secretary, Dr. Frank Trester Smith, of Chattanooga, Tenn., is actively at work preparing the program and all communications bearing on the subject should be addressed to him.

---

The Mississippi Valley Medical Association will hold its next annual meeting at Nashville, Tenn., October 11-14, 1898. The attendance promises to be large. Dr. John Young Brown, of St. Louis, is the president and Dr. Henry E. Tuley, of Louisville, is the secretary. The latter should be addressed at 111 West Kentucky street, in relation to places on the program or other details. Dr. George Ben Johnston, of Richmond, Va., will deliver the address on surgery and Dr. James T. Whittaker, of Cincinnati, that upon medicine.

---

The American Academy of Railway Surgeons will hold its fifth annual meeting at the Auditorium, Chicago, Ill., Wednesday, Thursday and Friday, October 5, 6 and 7, 1898, under the presidency of Dr. R. Harvey Reed, of Rock Springs, Wyoming. An elaborate program has been prepared by the secretary, Dr. D. C. Bryant, of Omaha, Neb., and an excellent meeting is in prospect.

---

The New York State Association of Railway Surgeons will hold its eighth annual meeting at the Academy of Medicine in New York City, November 17, 1898, under the presidency of Dr. C. B. Herrick, of Troy. The secretary is Dr. George Chaffee, of 226 47th street, Brooklyn, to whom all communications concerning the meeting should be addressed.

---

The American Microscopical Society, at its recent meeting at Syracuse, N. Y., elected the following-named officers for the ensuing year: President, Dr. William C. Krauss, of Buffalo; first vice-president, Professor A. M. Bleile, of Columbus, Ohio; second vice-president, Dr. G. C. Huger, of Ann Arbor, Mich.; secretary, Professor Henry D. Ward, of Lincoln, Neb.; treasurer, Magnus Pflaum, of Pittsburg; executive committee, Professor S. H. Gage, of Ithaca, N. Y.; Dr. A. Clifford Mercer, of Syracuse, and Dr. V. A. Moore, of Ithaca.

The Medical Association of Central New York will hold its next annual meeting at Auburn on Tuesday, October 18, 1898, under the presidency of Dr. William C. Krauss, of Buffalo. The secretary is Dr. C. A. Van der Beek, of Rochester, who should be addressed in regard to the program or other details concerning the meeting.

---

The New York State Medical Association will hold its next annual meeting at New York City, October 18, 19 and 20, 1898. Dr. Douglas Ayres, of Fort Plain, is president and Dr. E. D. Ferguson, of Troy, is secretary.

